# DNA-Based Diet Analysis for Any Predator

**DOI:** 10.1371/journal.pone.0005252

**Published:** 2009-04-23

**Authors:** Glenn Dunshea

**Affiliations:** 1 Antarctic Wildlife Research Unit, School of Zoology, University of Tasmania, Hobart, Tasmania, Australia; 2 Australian Marine Mammal Centre, Australian Antarctic Division, Kingston, Tasmania, Australia; American Museum of Natural History, United States of America

## Abstract

**Background:**

Prey DNA from diet samples can be used as a dietary marker; yet current methods for prey detection require *a priori* diet knowledge and/or are designed *ad hoc*, limiting their scope. I present a general approach to detect diverse prey in the feces or gut contents of predators.

**Methodology/Principal Findings:**

In the example outlined, I take advantage of the restriction site for the endonuclease *Pac I* which is present in 16S mtDNA of most Odontoceti mammals, but absent from most other relevant non-mammalian chordates and invertebrates. Thus in DNA extracted from feces of these mammalian predators *Pac I* will cleave and exclude predator DNA from a small region targeted by novel universal primers, while most prey DNA remain intact allowing prey selective PCR. The method was optimized using scat samples from captive bottlenose dolphins (*Tursiops truncatus*) fed a diet of 6–10 prey species from three phlya. Up to five prey from two phyla were detected in a single scat and all but one minor prey item (2% of the overall diet) were detected across all samples. The same method was applied to scat samples from free-ranging bottlenose dolphins; up to seven prey taxa were detected in a single scat and 13 prey taxa from eight teleost families were identified in total.

**Conclusions/Significance:**

Data and further examples are provided to facilitate rapid transfer of this approach to any predator. This methodology should prove useful to zoologists using DNA-based diet techniques in a wide variety of study systems.

## Introduction

An established field of dietary analysis is the use of prey DNA in the digestive system or feces of predators for prey identification [1], [2]. Ingested food contains species-specific DNA sequences, thus remnant DNA provides an excellent means of detecting and identifying material from prey [2]. Polymerase chain reaction (PCR) detection of prey DNA has proven effective when applied to both sacrificed invertebrate predators [3]–[5] and scats from vertebrate predators [6]–[8]. DNA-based methods offer the ability to identify prey where prey hard parts survive the digestion process differentially or not at all. The latter being the case for many invertebrate systems [2], for feces of particular vertebrate predators such as cetaceans and sea-birds [9] and with prey items with few hard parts. There are advantages over techniques such as lethal sampling and stomach lavage for hard part analysis, as DNA analysis of scat is non-invasive [10] and it has also been proven more sensitive and less variable than scat hard part analysis [11]. Development of DNA-based prey assays can be achieved relatively rapidly, compared to techniques utilizing monoclonal antibodies [12] and multiple prey items can be screened for simultaneously [e.g. 4]. These assays also offer greater taxonomic resolution of prey, though over shorter timescales, than methods such as fatty acid and stable isotope analyses [though see 13].

DNA extracted from diet samples is inevitably highly degraded [14], [15] and primarily consists of templates not relevant to diet studies (i.e. predator or gut fauna DNA) [8]. Most previous DNA diet studies have therefore targeted a small multi-copy DNA fragment (e.g. mitochondrial DNA (mtDNA) or nuclear ribosomal DNA) to increase the likelihood of successful PCR amplification [2] in conjunction with species- or group-specific PCR primers, which don't anneal to and amplify predator and gut fauna templates [e.g. 4], [8]. These assays score the presence of prey items by a successful PCR amplification, indicating a prey DNA template is present [e.g. 4]. Group-specific methods may also further identify prey by cloning PCR products and identifying clone amplicons via sequence analysis [8] or by some other amplicon separation and scoring method (e.g. denaturing gradient gel electrophoresis (DGGE); [6], [16]). These approaches are advantageous for targeted questions about specific prey taxa or study systems [e.g. 10], however they have obvious limitations for addressing broad diet questions or application outside of their original study system. The major drawback is that they assume *a priori* knowledge of diet and will overlook prey items in predators with diverse or uncharacterised diets [3]. Applying group/species-specific methods in many situations may also require considerable methodological development, as at present assays have only been designed for a limited number of prey groups or species [e.g. 4], [7], [17], [18], may not be useful to identify lower taxa [e.g. 8] or are only applicable to specific predators [6], [19], [20].

A PCR strategy that targets diverse items and is transferable across study systems would be desirable in many situations. Such an approach is reliant on the use of more or less ‘universal’ PCR primers; that is, PCR primers designed to anneal to target templates in as wide a range of taxa as possible [8 and references therein]. The use of universal primers on DNA extracted from samples with a dominant DNA template (i.e. diet samples) results in primarily the dominant template being amplified, which may not be of interest [6], [21], [22]. Employing universal primer analyses in these situations requires further manipulation of the DNA to exclude unwanted templates. Such approaches have been reported, but they may not be applicable outside of the system they were designed for without considerable further *in silico* and laboratory development [3], [22]–[24]. Consequently, like prey-specific approaches, the formulation of these universal methods was largely *ad hoc* and they are not immediately widely applicable. Furthermore, most of these approaches were based on PCR amplification of DNA regions >700 base pairs (bp) in length. The ability to successfully amplify DNA from diet samples in general, but particularly from DNA extracted from scat, is heavily dependent on the PCR target fragment (amplicon) size [14], [25]. For example, [14] found that only 1–19% of prey DNA extracted from sea lion scat samples was 226 bp in length and that in most instances <2% of prey DNA was >500 bp in length. Thus using universal primers for diet analyses requires as small a target fragment as possible, yet one which displays sufficient inter-specific variation for DNA-based identification.

Here I present a molecular method to detect diverse prey DNA in scats from most representatives of toothed whales (Odontoceti). It excludes predator DNA from novel universal primers using a restriction enzyme, leaving prey DNA intact for amplification and further analysis. This method differs from existing methods as an entire laboratory protocol immediately transferable to many predators (most odontocete's) is provided and it is applicable to entirely different study systems by changing only the restriction enzyme employed. More specifically this study: 1) Provides universal PCR primers for a small DNA fragment (essential for DNA-based diet work) which has been shown suitable for species level identification in most instances. The primers amplify a wide range of taxa and are thus immediately applicable across many study systems for vertebrate and invertebrate predators; 2) Provides sequence data for the same DNA fragment from 12412 species of animals which facilitates both rapid ascertainment of a suitable restriction enzyme to exclude predator DNA, as well as the ability to estimate the likelihood of exclusion of potential prey taxa. If the predator 16S sequence is not represented or recently added to GenBank, all that is required is to sequence the amplicon region of the predator and analyse it with the dataset provided in this study; 3) Provides evidence that the protocol employed here is effective at excluding predator DNA and identifying diverse diet items using bottlenose dolphins as an example, and; 4) Provides examples of diverse marine predator groups where this method can be immediately applied. The aims of this study were to present the rationale and proof of concept for the methodology along with the necessary data and framework to develop the method for any predatory group, with examples from apex marine predators.

## Methods

### *In silico* development

Widely conserved primers for arthropods through chordates were designed and tested for suitability *in silico* and empirically (Supporting Information S1) for a short (≈190–260 bp) region of 16S mtDNA within a larger region that has been used for DNA-based identification in other studies [26], [27] and using more selective primers [6], [19]. This shorter region proved generally suitable for DNA-based identification when examined using sequence data from GenBank (Supporting Information S1). Amplicon software [28] was used for primer design with one hundred 16S sequences selected randomly from GenBank across all chordates and from Echinodermata, Mollusca, Crustacea and Insecta. The primers contained degeneracy so an equal concentration mixture (i.e. equal volumes of each primer) of relevant forward primers was used in addition to the degenerate primer (5′-3′): Forward (16SPLSUFwd): AAGACCCTGTGGAGCTT, AAGACCCTATAAAGCTT, AAGACCCTATGGAGCTT, AAGACCCTGCGGAGCTT, AAGACCCTAATGAGCTT, AAGACCCTATAGAGCTT, AAGACCCTRHDRAGCTT. Reverse (16SPLSURv): RRATTRCGCTGTTATCCCT, RRATCRYGCTGTTATCCCT. The amplicon region was mapped for restriction sites across odontocete species on GenBank using BioEDIT [29]. The homologous restriction site for the 8-cutter enzyme *Pac I* was conserved across most of the examined odontocetes (see results). All available mammalian 16S sequences were then downloaded from GenBank in ordinal and sub-ordinal groups, aligned using MUSCLE [30], the fragment flanking sequences trimmed, alignment gaps removed and individual species scored for *Pac* I restriction site presence in the amplicon region using BioEDIT. Presence of the restriction site was similarly scored within the amplicon region of selected species groups across most animals represented on GenBank (As above; Table 1) to estimate any biases introduced by non-intentional exclusion of potential prey. All sequence alignments (of 12 412 species grouped by major lineage) are provided in Supporting Information S2.

**Table 1:** Proportions of species that contain the *Pac I* restriction site within the amplicon region out of species represented on GenBank (as of September 2006).

Group	Number of species in alignment	Taxa with *Pac I* recognition site (%)
**Mammalia**		
Theria		
Afrotheria	22	14
Euarchontoglires	303	8
Laurasiatheria		
***Carnivora***	86	79
***Microchiroptera***	265	75
***Odontoceti***	33	79
All other Laurasiatheria	257	25
Xenarthra	9	22
Metatheria	80	4
**Aves**	580	0
**Reptilia**	1568	1
**Amphibia**	456	<0.5
**Ray-finned Fish**	3306	1
**Other Chordates**	38	0
**Echinodermata**	198	<0.5
**Mollusca**	1288	1
**Crustacea**	1462	12
**Insecta**	2344	27
**Other Invertebrates**	116	11

All alignments are available in Supporting Information S2.

### Development of laboratory protocol

Scat samples (*n* = 5 from three different individuals; Table 2) were collected from captive *T. truncatus* housed at the Sea World Aquarium, Gold Coast, Australia. The study animals were fed a daily diet consisting of 15% wet weight *Scomber australasicus, Arripis georgianus* and *Mugil cephalus,* 10% *Nototodarus spp.,* 2% Penaeidae shrimp (‘constant species’) and the remaining 43% a variable amount and composition of *Trachurus novaezelandiae, Sillago robusta, Sardinella lemuru, Sardinops sagax* and *Pomatomus saltatrix* (‘non-constant species’). Meals were of unequal composition, 7.5–16 kg dependent on the mass and age of the individual dolphins and were fed at four scheduled daylight training sessions and intermittently during trainer interactions. Animals were housed in large pools of filtered sea water and were monitored during daylight hours from the pool perimeter by 1–2 observers. When defecation was observed the scat was collected as quickly as possible by running a net of nylon grit gauze, aperture 300 um on the end of a large pole through the fecal plume. Not all scats seen were able to be collected due to being out of reach or dissipating in the water column before being able to run nets through the fecal plume. Collected fecal material was washed out of the net into a plastic tray with 70% ethanol, poured into a sample jar with 70% ethanol (ethanol to scat ratio, 3∶1) and stored at 4°C until analysis. Plastic trays and nets were cleaned thoroughly between sample collections. All samples from captive animals used in this study were collected from known individuals, consisted of at least 5 cm^3^ of fecal material and were collected at least 48 hours after the known diet commenced.

**Table 2:** Prey and mammal clones identified in clone libraries from 5 captive feeding trial samples subject to Treatment 2 (*Pac I* digestion for predator DNA exclusion prior to PCR and also prior to cloning) with prey species abbreviations shown below.

Sample (Individual)	***Sa***	***Ag***	***Mc***	***N***	***P***	***Tn***	***Sr***	***Sl & Ss***	***Ps***	*Tt*	*Pg*	Clones Screened	Prey Species Detected
1(1)	11	5	0	2	0	1	0	32	0	0	1	52	5
2(2)	0	1	0	0	0	4	0	16	0	4	9	35	3
3(2)*	8	6	0	0	0	4	1	17	0	1	0	37	5
4(2)	2	13	16	0	0	0	0	2	0	0	5	38	4
5(3)	5	0	11	0	0	12	0	0	4	6	1	39	4
**Combined**	**26**	**25**	**27**	**2**	**0**	**21**	**1**	**67**	**4**	**11**	**16**	**201**	
**FOC**	**4/5**	**4/5**	**2/5**	**1/5**	**0/5**	**4/5**	**1/5**	**4/5**	**1/5**				
**Combined presence**	+	+	+	+	−	+	+	+	+				

* Sample 3 was collected 1.5 hrs after sample 2 on the same day from the same individual. *Sa: Scomber australasicus, Ag: Arripis georgianus, Mc: Mugil cephalus, N: Nototodarus spp., P: Penaeidae spp., Tn: Trachurus novaezelandiae, Sr Sillago robusta, Sl & Ss: Sardinella lemuru, Sardinops sagax, Ps: Pomatomus saltatrix, Tt: Tursiops truncatus,* Pg: *Tursiops truncatus pseudogene.*

Scat samples from five wild Sarasota Bay *T. truncatus* were collected in 2005 directly from the animal when they opportunistically defecated while being handled on deck of the research vessel for measurements and collection of other samples relevant to the Sarasota Dolphin Research Program's long term monitoring program [31]. Samples were stored for the day at 4°C until they were able to be fixed by addition of 100% molecular grade ethanol in the evening – ethanol was not available at the time of defecation. Samples were then stored at −20°C until further analysis. Each of these samples also consisted of between 5 cm^3^–15 cm^3^ of fecal material.

Fecal samples were homogenized by vigorous shaking and ≈1 ml of the ethanol/fecal slurry was aliquoted into DNA-free 2 ml tubes and centrifuged at 12 000g for 1 minute. The ethanol supernatant was removed and DNA was extracted from the fecal pellet using the QIAamp DNA Stool Mini Kit (QIAGEN) according to manufacturer's instructions for viral DNA extraction. Extractions were performed in a two batches (one for each sample set) with a blank (no starting material) extraction to monitor for cross-over contamination in each batch. Concentration of each DNA extract was <2 ng/µl as assessed by fluorometry using the PicoFluor system (Turner Biosystems) and PicoGreen dye (Molecular Probes). For the captive samples, sequences for the amplicon region of each prey item were sourced from GenBank for prey identification. Sequences were also generated and deposited for the remaining prey items not on GenBank for the captive feeding trial and for some likely prey items for Sarasota Bay dolphins. Here DNA was extracted from prey muscle tissue with a MoBio Tissue Extraction kit according to manufacturers instructions and 16S mtDNA PCR primers LR-J-12887 and LR-N-13398 and PCR reaction and thermocycling conditions from [32] were used to amplify a ≈620 bp fragment of 16S mtDNA. The fragment was purified and sequenced as indicated below for all other sequenced PCR products (GenBank accession: Feeding trial: EF590264, EF590265, Sarasota fish samples: EU239803–EU239814, EU239933).

To examine the efficacy of a *Pac I* restriction digest to remove predator amplicons, two treatments were applied to samples 1–4 from the captive trial; 1) restriction digestion of DNA prior to PCR then immediate cloning, and; 2) restriction digestion of DNA prior to PCR, then further restriction digestion of the PCR product prior to cloning. For the first treatment 34 µl of DNA from each scat was subject to *Pac I* (New England Biolabs) restriction digestion according to manufacturer's instructions, in a 45 µl total volume with 5 units of enzyme for 16 hours. The restriction enzyme was heat inactivated and a 16SPLSUFwd/16SPLSURv PCR performed with 2.5 µl of template DNA taken directly from the restriction digest reaction (see below). The PCR products were cloned, 24–28 clones directly amplified and sequences generated, all as described below. For the second treatment 34 µl of scat DNA was restriction digested as above, 2.5 µl of the digested DNA was subject to 16SPLSUFwd/16SPLSURv amplification (see below) and the PCR product purified using minelute spin columns (QIAGEN) as per manufacturers' instructions. The entire purified PCR product was further *Pac I* restriction digested according to manufacturer's instructions, for 4 hours with 5 units of enzyme in a 45 µl volume. The *Pac I* digested PCR product was then directly cloned, 35–52 clones directly amplified, SSCP screened and representative variant sequences generated all as described below. Sample 5 from the captive animals and all samples from free ranging Sarasota Bay dolphins had the latter treatment applied to them (see results). For the Sarasota samples, 17–20 clones were SSCP screened and representative sequences generated all as described below.

PCR reaction conditions for all 16SPLSUFwd/16SPLSURv PCR's were: 0.4 µM 16SPLSUFwd and 0.4 µM 16SPLSURv primer mixes, 1× AmpliTaq® Gold Buffer (Applied Biosystems), 2 mM MgCl^2+^, 1× BSA (New England Biolabs), 100 µM dNTP's (New England Biolabs), 0.75 units AmpliTaq® Gold DNA polymerase (Applied Biosystems), 0.05× SYBR® Green I (Molecular Probes) and the described amount of template for each 16SPLSUFwd/16SPLSURv PCR in a 25 µl total volume. PCR thermocycling conditions were an initial denaturation at 95°C for 7.5 minutes followed by repeated cycles (details below) of 95°C for 15 seconds, 52°C for 45 seconds and 72°C for 45 seconds. Scat PCR amplifications were conducted on a Real Time PCR thermocycler and associated software (Chromo4™ detection system: MJ research) and stopped within the exponential phase (usually between 15 and 25 cycles) in order to minimise PCR drift [33]. PCR of the blank DNA extractions yielded no amplification signal over 35 Q-PCR cycles as did PCR negative controls. After thermocycling all 16SPLSUFwd/16SPLSURv PCR's from scat DNA were incubated at 72°C for 20 minutes. PCR products were cloned using half reactions of the TOPO TA cloning system (Invitrogen) with vector pCR 2.1. Transformants were picked into 50 ul of ultra-pure water and plasmid DNA liberated by heat lysing.

Single strand conformation polymorphism (SSCP) analysis was used to score clones with identical inserts [34] to reduce sequencing redundancy. Here clones were 16SPLSUFwd/16SPLSURv PCR amplified using 5 ul of boil-lysed plasmid template and reaction conditions as described above with 35 cycles of thermocycling and a final 10 minute extension at 72°C. SSCP non-denaturing polyacrylamide gels (12 cm by 8 cm) were cast using 1× MDE® (BMA, Rockland, Maine) and 0.5× TBE according to manufacturers instructions. Size standards and 10 µl of 16S PCR product from each clone were denatured for 3 minutes at 95°C in one equal volume of formamide stop solution (95% formamide; 10 mM NaOH; 0.25% bromophenol blue, 0.25% xylene cyanol) and subject to electrophoresis at a constant wattage (6W) for 12 hours in 0.5× TBE at 15°C on the Bio-Rad DCode™ mutation detection system with a cooling bath attached. Run gels were stained in 200 ml 0.5× TBE, 50% glycerol, 0.5× SYBR® Gold (Molecular Probes) for 20 minutes and photographed. Photos representing each clone library were analysed for clones with identical banding patterns visually and using Image J software.

To sequence representative variant plasmid inserts from each clone library, plasmid pCR® 2.1 specific primers were used PCR plasmid inserts and these were directly sequenced. Here vector pCR® 2.1 specific primers (5′-3′: TOPO_F: GCCGCCAGTGTGATGGATA and TOPO_R: TCGGATCCACTAGTAACG) were used to amplify the plasmid insert of each unique clone using 5 ul of boil-lysed plasmid template in identical primer and reagent concentrations as for the 16SPLSUFwd/16SPLSURv PCR. Thermocycling was 95°C for 7.5 minutes followed 35 cycles of 95°C for 15 seconds, 52°C for 45 seconds and 72°C for 45 seconds and a final 10 minutes extension at 72°C. All PCR products intended for direct sequencing (both plasmid pCR® 2.1 specific PCR's of variant clones and prey muscle 16S mtDNA PCR's) were purified by isopropanol precipitation [35] and sequencing was carried out using a commercial service (Macrogen Inc.).

### Data analysis

Sequences recovered from clone libraries were edited, primer sequences removed and examined against reference sequences for the captive feeding trial samples or the GenBank nucleotide database using the BLAST algorithm [36] for the Sarasota samples. For both the feeding trial and the free-ranging samples, identity into a prey ‘operational taxonomic unit’ (OTU) was assigned to recovered scat sequences that had ≥98% similarity to prey sequences. This level of similarity allows for both *Taq* polymerase error and intra-specific variation [8]. Recovered sequences <98% similar to the closest prey match were assigned to familial OUT's based on their topographical position relative to taxonomic lineages in neighbor joining bootstrapped consensus trees constructed in MEGA 3.1[37] using closely matching sequences from BLAST analyses. All GenBank sequences available from the assigned familial OTU were then used to construct further similarity trees (as above) to examine whether the clone could be assigned to a lower OTU. Because chimeric DNA sequences can be obtained when PCR amplifying mixed, degraded DNA sources, all sequences unable to be identified to a low taxonomic level were analysed with chimera detection software (Ccode; [38]) against other identified sequences from the same clone library. Although nuclear mitochondrial pseudogenes (NUMTs) can confound prey identification using mtDNA [39], sequences <98% similar to the closest prey match were all most closely matching to teleosts and thus were not examined for NUMT origin, given the conspicuous absence of NUMTs in this chordate group [40], [41]. To examine the effect of restriction digestion treatment applied to samples on the proportion of predator amplicons recovered, proportions of predator and prey clones across samples within each treatment were pooled and treatments were tested for differences using chi-squared contingency tables with Yates correction.

## Results

### Taxon specific presence/absence of Pac I restriction site in amplicon

Of the 1056 different putative mammalian 16S mtDNA sequences containing the region of interest on GenBank, the *Pac I* restriction site was present in 75–79% of Carnivora, Odontoceti and Microchiropteran mammals (Table 1). The prevalence of the restriction site in other mammalian groups ranged from 8–25% (Table 1). In Odonotoceti all Physeteridae lacked the *Pac I* restriction site, as did *Globicephala melas*, *Inia geoffrensis, Monodon monoceros, Pontoporia blainvillei* and *Tasmacetus shepherdi*. In other Chordates, 5948 different species had putative 16S amplicon sequences and a range 0–1% of species contained the *Pac I* restriction site, dependent on the group (Table 1). A further 5408 species of invertebrates examined had putative 16S amplicon sequences; a range of 1–27% of these species contained the *Pac I* restriction site within the amplicon region (Table 1). Within the invertebrates, echinoderms and molluscs had little incidence of the *Pac I* restriction site in the amplicon region (<2% of all species examined), however there was a marked increase of the restriction site in the arthropods (12% of crustaceans and 27% of insects; Table 1).

### SSCP scoring of identical clones

**Figure 1:**
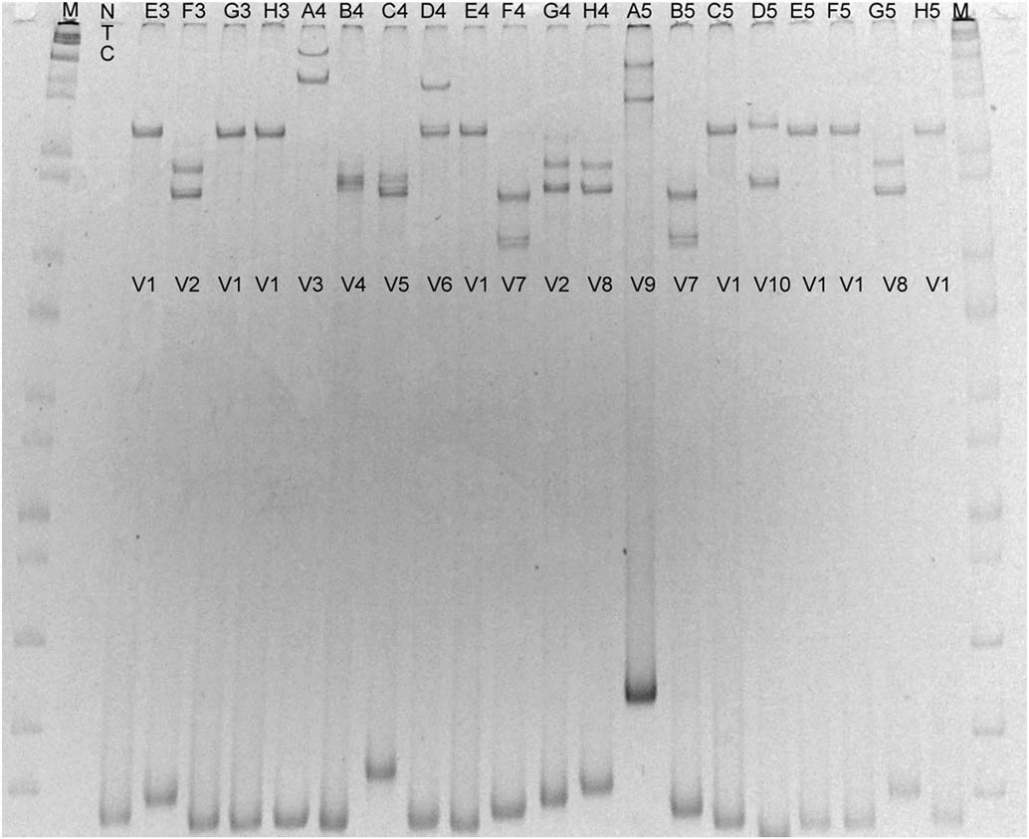
Example of SSCP gel used to identify identical clones from a clone library. M = molecular size marker, NTC = No template control of PCR, E3, F3… = Different clones by well position in 96 well plate. V1, V2… = Variant clones (i.e. All V1 clones are identical). Clone identities confirmed by sequencing: V1: *Sardinella lemuru*/*Sardinops sagax*, V2: *Scomber australasicus*, V3: *Trachurus novaezelandiae*, V4: Chimera of *Trachurus novaezelandiae* and *Sardinella lemuru*/*Sardinops sagax* sequences, V5: *Tursiops truncatus*, V6: *Sardinella lemuru*/*Sardinops sagax*, 2 substitutions difference from V1, V7: *Arripis georgianus*, V8: *Scomber australasicus* 1 substitution difference from V2, V9: Poor sequence – discarded, V10: *Sillago robusta*.

The SSCP banding patterns produced by this protocol could discriminate between DNA sequences differing by 1–2 base pairs (e.g. Fig 1. variant clones V1 & V6 and clones V2 & V8). Given this level of resolution, different banding patterns were discovered that belonged to the same prey OTU by sequence analysis. Because all variant clones sampled from each clone library were sequenced, these discrepancies could be identified.

### Removal of predator amplicons

The proportion of predator amplicons recovered from samples subject to treatment 1 where scat DNA was digested with *Pac I* prior to PCR and cloning ranged between 21–50%. The proportion of predator amplicons was further reduced to 2–37% when treatment 2 (*Pac I* digestion before and after PCR) was applied to the same samples. Pooling results across samples in each treatment, the reduction in predator amplicons in treatment 2 was significant (*x^2^* = 20.75, *d.f.* = 1, p = <0.001). Many of the few sequences of predator origin that were recovered from treatment 2 did not contain the *Pac I* restriction site, or display strong similarity to *T. truncatus* 16S mtDNA and have been identified as *T. truncatus* nuclear mitochondrial pseudogenes (‘NUMTs’) [39].

### Prey detection from captive dolphin scat

The number of prey OUT's identified per captive sample scat was similar regardless of treatment (range: 3–5). Pooling results from all samples resulted in 6 and 8 out of 9 prey OTU's detected in treatments 1 and 2 respectively. Although 10 species were present in the diet, *Sardinella lemuru* and *Sardinops sagax* shared an identical target fragment sequence so these species are effectively one OTU. All but one prey sequence detected shared at least 98% identity to prey DNA. In one instance an amplicon was identified as a chimera consisting of differing sequence regions from two prey items in scat 4 (Fig. 1; Table 2). Since Treatment 2 reduced incidence of predator amplicons, further results are presented for treatment 2 only. Pooling results from all samples subject to treatment 2, all prey except Penaeidae were detected (Table 2). In terms of frequency of occurrence of the ‘constant’ species, two of the prey items fed at 15% wet weight of the diet were detected in 4 of 5 scats and another detected in 2 of 5 scats, one item fed at 10% wet weight of diet was detected in 1 of 5 scats and an item fed at 2% wet weight of diet was not detected (Table 2). At least one ‘non-constant’ species was detected in all samples. Two non-constant prey OTU's were detected in 4 of 5 scats and the remaining two non-test OTU's were detected in 1 of 5 scats (Table 2). Two additional prey OTU's not detected in sample 2 were detected in sample 3; sample 3 was collected 1.5 hours after sample 2 from the same individual on the same day (Table 2). Mean diversity of prey OTU's identified per scat using treatment 2 was 4.2+/−0.84 (S.D.).

### Prey detection from free ranging Sarasota Bay dolphin scat

Treatment 2 was applied to Sarasota Bay dolphin scat samples since it reduced incidence of predator amplicons. In most samples few amplicons of predator origin were detected (0–5% of all amplicons in four samples), however in one sample 71% of amplicons assayed were of predator origin indicating little prey DNA was present in the sample (Table 3). Prey detected consisted of 13 different OTU's from 8 different families of bony fish (Table 3). Of the 13 different OTU's identified, 11 were assigned to species that displayed at least a 98% sequence similarity and in eight cases clone sequences were 100% identical to those obtained from GenBank. The two ambiguous cases were assigned to familial and generic level on the basis of their topographical position within ordinal/subordinal or familial distance-based neighbour joining phylogenetic trees (Table 3). The most important prey species in terms of frequency of occurrence were *Leiostomus xanthurus* (in all five scat samples), *Lagodon rhomboides* (in four of five scat samples) and *Cynoscion nebulosus, Mugil cephalus* and *Opsanus beta* (each in two of five scat samples). All other prey OTU's were unique to a single scat. Between 3 and 7 prey OTU's were identified per scat with a mean diversity of prey OTU's of 4.6+/−1.5 (S.D.).

**Table 3:** Prey operational taxonomic units (OTU's) identified from scat samples obtained from free-ranging Sarasota Bay *T. truncatus* (*n* = 5 different individuals).

Family	Prey OTU	Scat 1	Scat 2	Scat 3	Scat 4	Scat 5	FOC#
Batrachoididae	*Opsanus beta* ∧	X	X				**2/5**
	*Opsanus* spp*		X				**1/5**
Elopidae	*Elops saurus* ∧				X		**1/5**
Ephippidae	*Chaetodipterus faber* ∧		X				**1/5**
Hemiramphidae	*Hemiramphus brasiliensis* ∧	X					**1/5**
Mugilidae	*Mugil cephalus*	X				X	**2/5**
	*Mugil curema* ∧					X	**1/5**
Paralichthyidae	*Paralichthys albigutta* ∧			X			**1/5**
Sciaenidae	*Cynoscion nebulosus* ∧		X		X		**2/5**
	Sciaenidae spp**			X			**1/5**
	*Leiostomus xanthurus*	X	X	X	X	X	**5/5**
Sparidae	*Archosargus probatocephalus* ∧		X				**1/5**
	*Lagodon rhomboides*	X	X	X	X		**4/5**
**Prey sp. per scat**		**5**	**7**	**4**	**4**	**3**	
**Predator amplicons (%)**		**0**	**5**	**5**	**0**	**71**	

∧ Sequences recovered from all scat clone libraries were 100% identical to sequences of these species from GenBank.

* Grouped with *Opsanus* spp in bootstrapped consensus NJ tree of order Batrachoidiformes with 96% bootstrap support, closest BLAST match: *Opsanus tau* 92% identity over whole clone sequence.

** Grouped with Sciaenidae in bootstrapped consensus NJ tree of suborder Percoidei with 20% bootstrap support, closest BLAST matches: *Sciaenops ocellatus*, *Cynoscion leiarchus* and *Cynoscion microlepidotus* - 91% identity over 98% of clone sequence.

# FOC: Frequency of Occurrence.

## Discussion

The method presented here was successful in identifying prey DNA in dolphin scats from 14 different teleost families and 1 cephalopod. All but one prey group fed as a small proportion of the overall diet were detected from the known diet samples. The primers used in this study successfully amplify 16S mtDNA from a range of higher taxa (see Supporting Information S1 for analysis of priming regions) and the amplified fragment generally has adequate sequence characteristics to identify lower taxa (Supporting Information S1). The restriction enzyme *Pac I* used to exclude predator mtDNA was present in the amplicon region of most representatives of the Carnivora, Odontoceti and Microchiroptera but largely absent in most other higher taxa, facilitating the exclusion of most predator DNA in these taxa. Specifically for odontocetes, the *Pac I* restriction site was largely absent in relevant (i.e. aquatic) prey taxa.

### Captive Samples

All but one prey OTU were identified from the captive feeding trial samples, although, at best, just over half of the prey OTU's were identified in any single scat. This is unsurprising given the uncontrolled nature of this feeding trial; specifically: 1) Not all scats produced by the study animals were collected (i.e. those not seen or able to be collected during poolside monitoring or produced at night); 2) Only a small proportion of any single scat was collected; 3) It is unsure what prey should be present in any given scat since the specific timings of prey ingestion is unknown for specific prey species and 4) it is not known how variable prey detection is when applying DNA methods to cetacean scat. There is evidence for meal specific pulses of a particular prey's DNA signal in pinniped scat [6], [11] and that detection rates of different prey species may differ [e.g. 11], both of which may have influenced prey detection in this case, particularly as meals of variable composition were fed four or more times throughout the day. That two additional species (as well as those detected previously) were detected in samples from the same animal taken 1.5 hours apart (captive samples 2 and 3) suggests prey DNA may also be passed in meal-specific pulses in *Tursiops truncatus*.

The fact that 9 out of 10 prey OTU's in a diverse diet representative of a generalist forager were identified from so few samples is encouraging. Five known primary prey items were identified in a single scat in this study. Other studies on captive vertebrates employed either species-specific [9], [11], [42] or group-specific primers [6], [11] and the maximum number of species identified in a single scat has been four [6]. These studies were able to collect entire bowel movements, generally fed their predators of interest (all pinnipeds) less prey taxa and only targeted relatively few of the fed prey items for PCR [e.g. 11], [42]. A potential pitfall in analysing the captive samples was the potential for prey DNA to be present in the water column and collected along with the scat. Unfortunately water samples were not collected and analysed, which would have controlled for this possibility. This is potentially a serious problem facing studies that propose to sample scat out of the water column [1] and could have also biased the results of previous studies sampling from water.

### Free-ranging Samples

An example of the utility of this method to yield novel dietary insights is provided by the analysis of scat samples from Sarasota Bay wild *T. truncatus*. These data are the first to directly identify prey species of live free-ranging Odontocete cetaceans, without direct observation of feeding. The main prey species identified by frequency of occurrence during this study (*Leiostomus xanthurus*, *Lagodon rhomboides, Cynoscion nebulosus, Mugil cephalus* and *Opsanus beta*) have been previously reported as important prey species for this population [from stomach contents analysis; 43], however the diversity of prey items identified in this study from 5 samples rivals that reported previously from 16 stomach contents samples analysed by hard part analysis [43]. In addition, possible prey species not previously reported have been identified (*Hemiramphus brasiliensis* and *Paralichthys albigutta*) [43], [44]. Since these samples were collected outside of the water column, it is less likely that environmental contamination would have biased these results.

### Comparison with Other Techniques

A range of strategies for preferentially amplifying prey DNA with widely conserved primers have been tested. These include primers that have a mismatch with predator DNA at the 3′ end of the primer [6], [19], [20], restriction digestion of predator DNA and/or predator amplicons [This study; 3], and use of ‘blocking primers’ that bind specifically to predator DNA inhibiting annealing of one universal primer and from which DNA polymerase cannot extend [23], [45]. All of these techniques may result in some prey DNA sequences being inadvertently suppressed from amplification along with predator sequences. It is unlikely any technique can ever account for this given the constraints of working with small DNA fragments, incomplete databases and in some instances, the unpredictable reactivity of reagents with prey sequences. A major advantage of the method presented here over previously applied methods is the predictability of its effects on the range of prey sequences being targeted. Furthermore, this method is easily adaptable to new combinations of predator and prey. Conceptually similar studies, such as [3] used restriction enzymes on universal PCR products from gut contents to exclude predator amplicons. Their study restriction digested PCR products after a universal primer PCR whereas in this study DNA was digested before as well as after PCR, to facilitate initial amplification of the rarer (prey) templates that otherwise may have been inhibited or excluded by predator DNA amplification. Although similar in experimental approach, this method is not transferable to studies of other animal diets as the region targeted is too large to amplify reliably from scat DNA and the applicability of the restriction digestion system was not assessed for other situations. Blocking primers, in comparison, have been shown extremely effective at excluding predator DNA in some situations [23]. Yet they suffer from being specific to single predators, the need for extensive empirical testing and no clear design criteria that ensures they will be effective in blocking predator DNA and not block prey DNA. In contrast, restriction enzymes are a known quantity; as one of the oldest tools in molecular biology their specificity is well documented and their behavior predictable. Primers that are mismatched to predator DNA may also be mismatched to certain prey DNA and are generally not transferable to other systems.

This methodology has its advantages and disadvantages. The advantages are its transferability across study systems and identifiable biases, little need of *a priori* diet knowledge and importantly, the fact that little further laboratory development is required. As a disadvantage, it is a multi-step protocol and is more time-consuming and expensive compared to single PCR assays targeting specific prey taxa. It is thus applicable where general trophic links are to be defined and diet breadth is to be examined, though it is clearly prohibitive to apply to thousands of samples in its current form. There may be scope to refine the protocol further to remove the cloning steps using other large scale amplicon diversity assays such as pooling PCR products and performing massively parallel sequencing [45]. Nevertheless this protocol offers a first approximation of trophic links to facilitate development of more specific assays, [such as those of 4], which is no trivial task in light of cross-reactivity issues between prey taxa [1]. Specific assay development and underlying assumptions are best guided by empirical data. This method therefore offers an ‘orientation’ assay in novel study systems and a useful complement to specifically targeted assays. An unexpected consequence of excluding predator mtDNA with a restriction enzyme was the appearance NUMTs in clone libraries, however blocking primer or hybridization approaches appear to suffer from the same issue [23], [45]. In many circumstances NUMTs can usually be recognised as such by employing simple comparative analyses. Additionally, it may be possible to use a further selective predator-specific restriction enzyme to exclude NUMTs from amplification without excluding prey (G Dunshea, *unpublished data*).

### Unintentional Prey Exclusion

This exact protocol could be applied to many carnivore or microchiropteran mammals, given the high incidence of the *Pac I* site in the amplicon region of these taxa. However, there is the potential for unintentional exclusion of prey taxa that also have the *Pac I* site such as other carnivores and laurasiatherians (25% represented on GenBank), within mammals and some Crustacea (12%) and Insecta (27%) within invertebrates, or for example, when applying to an odontocete that preys on another odontocete or carnivore (e.g. *Orcinus orca*). Yet by changing the restriction enzyme employed given the specific predator, or using different enzymes and mixing products prior to making clone libraries, these issues can be overcome. If interested in the insect diet of a microchiropteran, the restriction site for the enzyme *Avr II* is present in 100% of microchiropterans but no insects represented on GenBank. Similarly, if the mammalian diet of a carnivore is of interest, enzymes such as *Bcu I*, *Nde I* or *Mfe I* may be used, all of which have a restriction site in 100% of carnivores but no other mammals represented on GenBank. With the PCR primers and sequence alignments provided and the use of software for rapidly identifying restriction enzymes that cleave DNA regions in nominated groups but not in others [46], this method can be applied to any predator or predatory group from any taxonomic level with negligible and somewhat identifiable biases. Of course, the absence of the restriction site in a potential prey taxa represented in datasets does not guarantee that unknown taxa in that group are also without the restriction site. However, all universal methods more or less suffer the same constraint with their exclusion technique (i.e. are unable to account for reactivity with unknown diversity) and this method does at least allow for this issue to be unequivocally examined with available data. Moreover, using different restriction enzymes to exclude predator DNA may circumvent this issue, or similarly, using a different DNA region primer set [3], [19]. Further information on restriction enzymes to use for a variety of apex marine predators and an example of how this protocol may be applied to a specific predator (*Orcinus orca*) is provided in Table 4. Since this study was examining the diet of dolphins that consisted of fish, cephalopods and a crustacean known not to contain the *Pac I* restriction site (in the captive animal case) and most likely fish and possibly cephalopods in the free ranging animal case [43], the potential biases introduced by the *Pac I* assay were not of concern.

**Table 4:** Examples of restriction endonucleases that will cleave marine predator's DNA within the target 16S amplicon region but leave prey DNA intact for amplification and further analysis.

Marine Predator group	Predator Taxon (number of species analysed)	Endonucleases for 100% predator and 0% prey*^1^ amplicon exclusion
Seabirds*	Charadriiformes (72)	*Alw* NI; *Bst* 4CI; *Mva* I; *Pas* I; *Tsp* RI
	Pelecaniformes (9)	*Bse* 1I; *Pas* I; *Tsp* RI
	Procellariiforms (6)	*Pas* I
	Sphenisciformes (18)	*Mfe* I; *Pas* I
**Fur Seals** * **and Sea lions** *	Otariidae (8)	*Bc* 1I; *Bsa* BI; *Nmu* CI; *Xba* I
**Sperm Whales** *	Physeteroidea (3)	*Bc* 1I; *Eco* 147I; *Hpy* 188I
**Killer Whales** * **^1^**	*Orcinus orca* (1)	*Hind* III; *Pce* I

* & ^1^: Prey taxa analysed (number of species): *Ray-finned Fish (3306), Echinodermata (198), Mollusca (1288), Crustacea (1462). ^1^ Other Odontoceti (32), Phocidae (18), Otariidae (8), all from S2.

### Conclusion

Recent reviews on DNA-based diet work have noted the need for initial amplification of diet samples with group-specific or ‘general’ primers in uncharacterised systems [1], [12], yet few strategies have been offered to apply general primers to diet samples. This is surprising as it is well established that using general primers on diet samples results in predominately only predator DNA being amplified [3], [6], [21]. There is potential for DNA-based methods to identify a wide variety of prey of generalist predators [11], though in practice there have been few studies to do so [e.g. 3], [4], [19]. Progress is hindered by the constraints of methodological development and the need for *a priori* knowledge of predator diet. This study has demonstrated the utility for prey identification of a method that can predominately exclude predator DNA from amplification by PCR primers that target both vertebrate and invertebrate groups. The exact method presented here is directly applicable to most toothed whales and, with the aid of data presented and methods used in this study, the method is applicable to any predator group with minimal adjustment. The method needs little *a priori* knowledge of diet and may be used to in conjunction with, or to aid in, designing more specific tests. It should prove useful for complementing other diet analysis approaches and in defining predator-prey interactions where more established approaches are not yet feasible.

## Supporting Information

Supporting Information S1 (0.65 MB DOC)

Supporting Information S2 (0.78 MB ZIP)
