# Supplementary material for: DNA-Based Diet Analysis for Any Predator
Source: PLoS One. 2009 Apr 23;4(4):e5252. doi: 10.1371/journal.pone.0005252 (PMC2668750; doi:10.1371/journal.pone.0005252)
Supplement: Supporting Information S1 — (0.65 MB DOC) [file pone.0005252.s001.doc]

DNA-based diet Analysis for Any Predator

*Glenn Dunshea^1, 2^*

^1^ Antarctic Wildlife Research Unit, School of Zoology, University of Tasmania, P. O. Box 252-05, Hobart, Tasmania 7005, Australia

^2^ Applied Marine Mammal Ecology Group, Australian Antarctic Division, 203 Channel Highway, Kingston, Tasmania, Australia, 7050. Ph: +61 3 6232 3145, Facsimilie: (+61)362323449

Email: [glenn.dunshea@aad.gov.au](mailto:glenn.dunshea@aad.gov.au)

**ELECTRONIC SUPPLEMENTARY MATERIAL**

**Supplement** **1a: Test of ‘universal’ primers designed in this study to amplify 16s mtDNA from a wide variety of taxa**

***Introduction & Methods***

*In Silico analysis*

To examine whether primers designed in this study were ‘universally’ complimentary to priming regions of target taxa, alignments in supplement 2 were used to assess whether the priming regions were exactly complimentary to the primers presented in the main manuscript (5’ – 3’ FWD: AAGACCCTRHDRAGCTT; RV: RRATTRCGCTGTTATCCCT, RRATCRYGCTGTTATCCCT). This analysis was conducted in BioEdit. Unfortunately for most taxa in these alignments, the priming regions had been trimmed such that 2-4 nucleotides at the 5’ end of the forward primer and 7-12 nucleotides at the 5’ end of the reverse primer were missing, limiting the accuracy of these analysis. Nevertheless, given the deliberately low annealing temperature of the PCR assay, the fact that primers with mismatches away from the 3’ end can still anneal and allow polymerase to extend and that G - T ‘wobble bonds’ were not accounted for, these figures are probably a reasonable approximation of the true annealing capacity of these primers, particularly given the results of the empirical testing (see below). Additionally, BLAST searches indicated that the reverse primer sequences were far more highly conserved than the forward primer sequences.

The degenerate forward primer provided in the manuscript is 32 fold degenerate (i.e. allows for 32 different nucleotide combinations in degenerate positions). Only the most 10 frequently occurring nucleotide combinations of this primer were included in this analysis. Since it was found that many taxa were not exactly complimentary to these 10 primers (see results), an additional nine forward primers not presented in the main manuscript were added to this analysis: AAGACCCCGTTGAGCTT, AAGACCCTGTCGAGCTT, AAGACCCTATCGAGCTT, AAGACCCTTTGGAGCTT, AAGACCCTATAAAACTT, AAGACCCTGTGGAACTT, AAGACCCTATCGAACTT, AAGACCCTATAAATCTT and AAGACCCTATAGATCTT. Each of these additional primers differed by 1-2 base pairs from the primer combinations in the degenerate forward primer (Table S1).

*Empirical testing*

To empirically test whether the mixes of ‘universal’ primers in the main manuscript would amplify 16s mtDNA from a range of taxa from chordates through to insects, DNA was obtained from representatives of most major animal lineages within this range of taxa (See Figure S1 caption). This DNA was used as a template (1ul) in a 25ul volume PCR with reaction conditions as described in the main manuscript. Thermocycling used was 95º C for 7.5 minutes followed 35 cycles of 95º C for 15 seconds, 52º C for 45 seconds and 72º C for 45 seconds and a final 10 minutes extension at 72º C. To visualise PCR products, 1ul of product from each reaction was subject to electrophoresis in a 1x T.A.E, 2% agarose gel for 30 minutes at 100 volts. The gel was subsequently stained with ethidium bromide and photographed under UV light.

***Results & Discussion***

*In Silico analysis*

The primers presented in the main manuscript were directly complimentary with most

mammals (98%) and ray finned fish (80%) and only 2-51% of other taxa in alignments of supplement 2 (Fig. S1.). Since this study is tailored for marine systems the lack of compliment with some major marine lineages was of concern. To examine the extent of non-complementarity of the original primers, additional forward primers were included in the analysis that slightly differed from the primers presented in the main manuscript (5’-3’): AAGACCCCGTTGAGCTT, AAGACCCTGTCGAGCTT, AAGACCCTATCGAGCTT, AAGACCCTTTGGAGCTT, AAGACCCTATAAAACTT, AAGACCCTGTGGAACTT, AAGACCCTATCGAACTT, AAGACCCTATAAATCTT and AAGACCCTATAGATCTT. When these additional primers were included in the analysis the exact complementarity for marine groups increased to 80-98%. Note that most of these additional primers all differ by 1 - 2 base pairs to the primers presented in the main manuscript (and particularly mostly away from the 3’ end of the primer and/or at positions that will allow G-T wobble bonds) (see Table S1) and the initial primers presented still appear to amplify DNA from representative of these taxa (see Table S1 and Fig. S2.). It may be advisable to include all 19 of these primers in an equal concentration degenerate mix for future investigations in novel study systems using this DNA region. Given this study was detecting cephalopods, fish and penaeid crustaceans that were known to be complimentary to the primers in the main manuscript, these results were not of concern.

*Empirical testing*

When applying only the primers presented in the main manuscript, an amplicon of the expected size was amplified in all taxa assayed (Fig. S2), including some taxa not containing an exact compliment to the forward primer (Fig S1, Table S1). Additional bands were present in some taxa (Fig. 1, lanes 9, 13, 16, 17, 18) indicating some non-specific amplification. In all taxa except a mussel (lane 13, Mollusca: Pteriomorpha sp.) the additional band could most likely be separated from the expected size product by electrophoresis and excising bands in the expected size range from an agarose gel. When this assay is applied to a predator which preys on taxa which create additional bands with these primers, it may be necessary to excise the expected size product from the agarose gel before further restriction digestion and cloning. This could be examined on a case by case basis by running out PCR products from a sub-sample of PCRs performed with diet sample derived DNA to assess whether additional bands are being generated.

**Figure S1.** Proportion of species in alignments provided in supplement 2 (number of species in each alignment provided in parentheses – x-axis) that have priming regions directly complimentary at 3’ ends to the primers presented in the main manuscript (grey bars). Hatched bars represent the same analysis when the additional primers (5’-3’): AAGACCCCGTTGAGCTT, AAGACCCTGTCGAGCTT, AAGACCCTATCGAGCTT, AAGACCCTTTGGAGCTT, AAGACCCTATAAAACTT, AAGACCCTGTGGAACTT, AAGACCCTATCGAACTT, AAGACCCTATAAATCTT and AAGACCCTATAGATCTT are included. Exact proportion is indicated above bars.

**
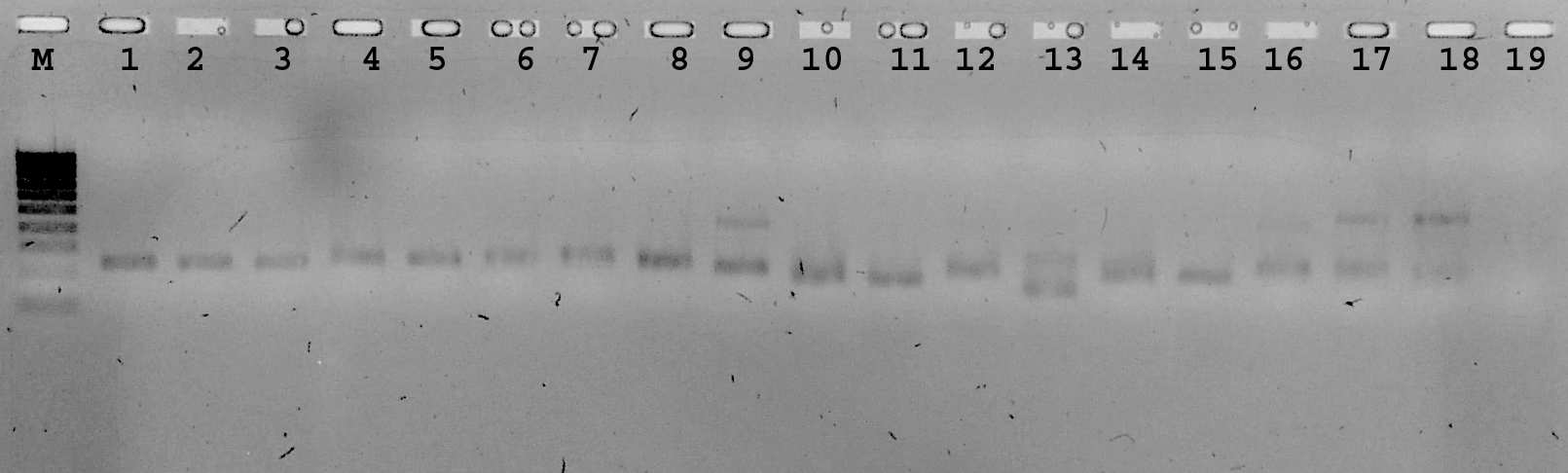
**

300bp

200bp

100bp

**Figure S2.** Test of universal primers designed for the small fragment of 16s mtDNA targeted in this study. Lanes: **M** : 1kb ladder; **1**: Mammalia: *Arctocephalus pusillus doriferus*, **2**: Mammalia: *Phoca groenlandica*, **3**: Mammalia: *Tursiops truncatus*, **4**: Aves: *Aptenodytes patagonicus*, **5**: Aves: *Stercorarius* sp. **6**: Teleosti: *Sardinops sagax*, **7**: Teleosti: *Trachurus novaezelandiae*, **8**: Echinodermata: *Centrostephanus rodgersii*, **9**: Echinodermata: Crinoidea sp. **10**: Mollusca: *Nototodarus* sp. **11**: Mollusca: *Nototodarus gouldi* **12**: Mollusca: Octopoda sp. **13**: Mollusca: Pteriomorpha sp. **14**: Crustacea: Amphipoda sp. **15**: Crustacea: Penaeidae sp. **16**: Crustacea: *Thysanoessa macrura*, **17** Insecta: Lepidoptera sp. 1, **18:** Insecta: Lepidoptera sp. 2. **19**: PCR no template control.

**Table S1.** Forward primers tested for 3’ complementarity with sequences provided in supplement 2. Primers 1-10 are present in the degenerate forward primer presented in the main manuscript and used in the study and primer pair empirical trial. Primers 11-19 are not represented within the degenerate forward primer in the main manuscript; nucleotide positions where these primers differ from those used in the study are shown in italicised, bold and larger font.

| Primer 1 | AAGACCCTGTGGAGCTT |
| --- | --- |
| Primer 2 | AAGACCCTATAAAGCTT |
| Primer 3 | AAGACCCTATGGAGCTT |
| Primer 4 | AAGACCCTGCGGAGCTT |
| Primer 5 | AAGACCCTAATGAGCTT |
| Primer 6 | AAGACCCTATAGAGCTT |
| Primer 7 | AAGACCCTATTGAGCTT |
| Primer 8 | AAGACCCTATAGAGTTT |
| Primer 9 | AAGACCCTGTTGAGCTT |
| Primer 10 | AAGACCCTATGAAGCTT |
| Primer 11 | AAGACCC***C***GTTGAGCTT |
| Primer 12 | AAGACCCTGT***C***GAGCTT^$^ |
| Primer 13 | AAGACCCTAT***C***GAGCTT |
| Primer 14 | AAGACCCTATAAA***A***CTT^$^ |
| Primer 15 | AAGACCCTGTGGA***A***CTT^$^ |
| Primer 16 | AAGACCCTAT***C***GA***A***CTT^$^ |
| Primer 17 | AAGACCCTATAAA***T***CTT |
| Primer 18 | AAGACCCT***T***TGGAGCTT |
| Primer 19 | AAGACCCTATAGA***T***CTT |

^$^ Indicates species with this forward primer binding site amplified with apparently similar efficiency as those with exactly complimentary binding site in empirical trial (Aves sp., Echinoderms, Pteriomorpha molluscs, amphipod crustaceans and *Thysanoessa macrura –*see Fig. S2).

**Supplement** **1b: Assessment of target 16SPLSU fragment to identify species from sequence data**

*Introduction & Methods*

To assess the suitability of target amplicon of 16s mtDNA in this study to identify species from sequence data, analyses were conducted similar to Hajibabaei et al. (2006) where within and between species divergence is compared; if within species divergence is equal to or exceeding between species divergence, then distance based DNA sequence identification would be ambiguous in that case. Familial (or the nearest higher taxon) from each potential prey higher taxon were selected and downloaded from GenBank that had the relative (compared to other familial units in the higher taxon) highest number of species within their familial (or nearest taxon) unit with 2 or more sequences from different individuals of each species. Only species with multiple individuals were included in each familial (or nearest taxon) unit. Downloaded sequences from familial (or nearest taxon) units were aligned using MUSCLE and flanking sequences trimmed in BioEDIT. The target fragment has two sites where indels are common and seemingly a diagnostic character in species identification. Therefore alignments were visually checked in these ambiguous regions and adjusted for obvious mistakes. These indels can be large when aligning distantly related taxa, however they were small when aligning familial (or nearest taxon) groups (<10 bp region). Aligned, trimmed and checked familial (or nearest taxon) units were imported into MEGA, individual sequences for each species were grouped and to assess divergence, genetic distance estimates within species and pairwise distance estimates between species were generated using the Kimura 2 parameter (K2P) model with pairwise deletion in indels regions. The distance estimates within species were compared to pairwise distance estimates between species and those species where within species distance equalled or exceeded a pairwise between species K2P distance were noted.

*Results & Discussion*

Table 1 summaries the taxonomic groups used for intra/inter-specific comparisons of genetic divergence as well as the number of species included, number of sequences per species, summary statistics of the K2P distance metrics and the proportion of comparisons where an intra-specific distance equalled or exceeded the compared pairwise inter-specific K2P distance with another species. All taxa except acanthurid and belonid teleosts had at least one species where intra-specific divergence equalled or exceeded a pairwise inter-specific divergence estimate (Table 1). In the vertebrate taxa there were relatively few overlaps between intra-specific and pairwise inter-specific distance estimates, excepting reptilians and epinephelid teleosts, where more pronounced overlap did occur between species (Table 1; Figure S3). Most overlap that did occur was when one or a few species had very high intra-specific distance estimate(s) compared to the rest of the familial (or nearest higher taxon) unit (Table 1). It is likely that the numbers of species where within and between species genetic divergence overlaps presented here as minimum estimates given that the taxa analysed are likely to not have all species sampled and the range of genetic divergence within a species is for the most part most likely underestimated.

**Table S2.** Summary of comparisons of within species divergence with between species divergence of species within each familial (or closest higher taxon) group.

| **Higher Group** | **Lower Group**  **(# of species included)** | ***X* (range)**  **sequencesper species** | ***X* WSD^1^ (range)** | ***X* BSD^2^ (range)** | **WSD ≥ BSD**  **(% of all comparisons)** | | ***X* (range) WSD in overlapping species** |
| --- | --- | --- | --- | --- | --- | --- | --- |
|  |  |  |  |  | **Intra-generic** | **Inter-generic** |  |
| **Amphibia** | Hylinae (38)  Mantellinae (71) Rhacophorinae (58) | 2.8 (2-7)  6.6 (2- 27)  5.8 (2- 42) | 0.019 (0-0.135)  0.049 (0-0.179)  0.038 (0.0.155) | 0.243 (0.005-0.467)  0.243 (0.004-0.462)  0.252 (0.027-0.384) | 0.1  0.2  0.1 | -  -  - | 0.019 (0.017-0.021)  0.135 (0.109-0.179)  0.067 (0.011-0.124) |
| **Aves** | Emberizinae (19)  Pardalotidae (15) | 2.1 (2-3)  2.3 (2-4) | 0.001 (0-0.005)  0.001 (0-0.008) | 0.075 (0-0.139)  0.053 (0.006-0.091) | 0.2  0.5 | -  - | 0.008 - |
| **Mammalia** | Vespertilionidae (32)  Hominoidea (16) | 5.1 (2-13)  17.6 (2-105) | 0.014 (0-0.066)  0.003 (0-0.016) | 0.228 (0-0.996)  0.207 (0-0.414) | 0.2  0.4 | -  - | 0  - |
| **Reptilia** | Chamaeleonidae (30)  Iguanidae (14) | 5.5 (2-29)  2.4 (2-5) | 0.022 (0-0.192)  0.018 (0-0.083) | 0.188 (0-0.368)  0.135 (0-0.329) | 1.1  3.3 | 1.8  - | 0.086 (0-0.192)  0.042 (0-0.083) |
| **Teleostei** | Acanthuridae (21)  Belonidae (26)  Epinephelinae (23) | 3 (2-4)  2.9 (2-8)  3.5 (2-27) | 0.003 (0-0.018)  0.008 (0-0.093)  0.012 (0-0.085) | 0.121 (0.017-0.23)  0.195 (0.005-0.345)  0.146 (0-0.454) | -  -  4.3 | -  -  0.2 | -  -  0.077 (0.068-0.085) |
| **Crustacea** | Aeglidae (48)  Parastacidae (60)  Penaeoidea (28) | 3 (2-9)  4.4 (2-30)  4.2 (2-16) | 0.006 (0-0.04)  0.013 (0-0.109)  0.006 (0-0.052) | 0.036 (0-0.088)  0.22 (0.01-0.455)  0.168 (0-0.313) | 2.4  0.9  0.4 | -  -  - | 0.018 (0-0.026)  0.065 (0.014-0.109)  0.005 (0.001-0.008) |
| **Insecta** | Diptera (110)  Lepidoptera (31) | 4 (2-46)  2.5 (2-6) | 0.011 (0-0.227)  0.008 (0-0.065) | 0.21 (0-0.444)  0.188 (0-0.314) | 0.1  0.4 | 0.6  - | 0.062 (0-0.227)  0.002 (0.-0.003) |
| **Mollusca** | Lampsilinae (12)  Sepiidae (16)  Mytiloida (6) | 6.2 (2-21)  2.9 (2-6)  16.7 (2-36) | 0.032 (0-0.163)  0.031 (0-0.16)  0.062 (0-0.14) | 0.134 (0-0.225)  0.166 (0.009-0.267)  0.275 (0.077-0.589) | 6.1  3.3  6.7 | 4.5  0.4  - | 0.07 (0-0.163)  0.111 (0.014-0.16)  0.084 (0.079-0.088) |

^1^ Within species Kimura 2 parameter genetic distance estimate

^2^ Between species Kimura 2 parameter genetic distance estimate


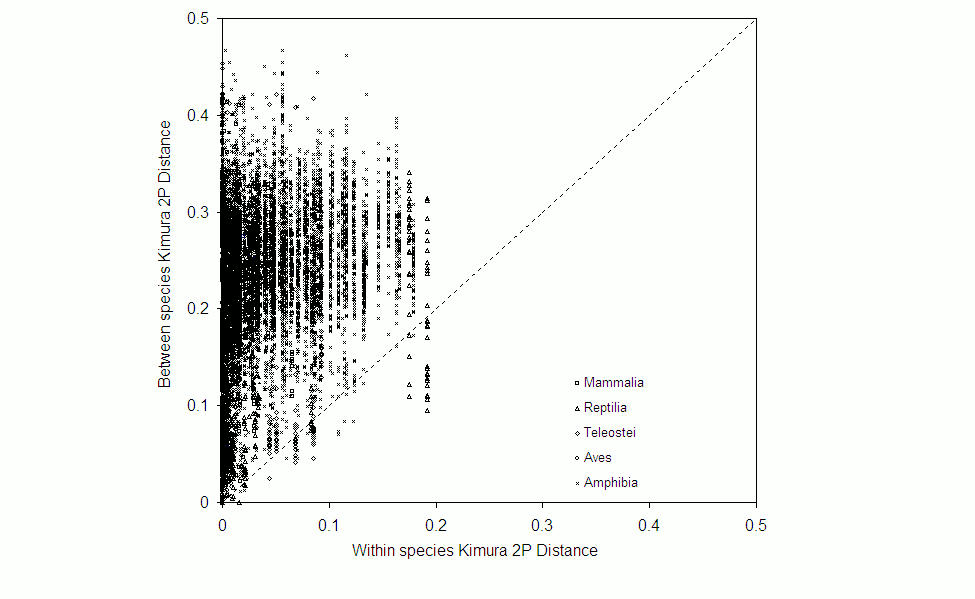


**Figure S3.** Comparison of within species K2P distance and between species K2P distance in vertebrate taxa from Table 1. Diagonal represents equal within and between species K2P distances. Points falling on or below diagonal line represent species that have equal or higher intra-specific divergence compared to the pairwise divergence with at least one other species. In such cases specific identification from sequence data based on distance measures would be ambiguous.


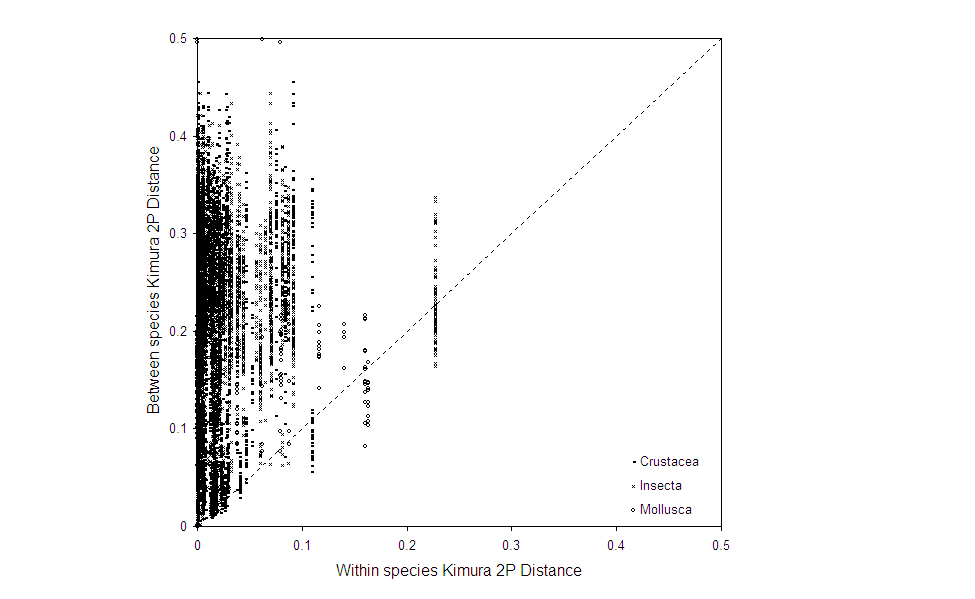


**Figure S4.** Comparison of within species K2P distance and between species K2P distance in invertebrate taxa from Table 1. Diagonal represents equal within and between species K2P distances. Points falling on or below diagonal line represent species that have equal or higher intra-specific divergence compared to the pairwise divergence with at least one other species. In such cases specific identification from sequence data based on distance measures would be ambiguous.

For invertebrates there was in general a greater overlap of intra-specific distances with inter-specific distances (Table 1; Figure S4). However, all inter-specific comparisons where intra-species divergence was ≥ inter-specific divergence remained under 7% of the total pairwise comparisons indicating that even though there will be greater ambiguity with invertebrate sequence based identification in general, the majority of occasions should still produce an unambiguous identification. Again, (excepting penaeid crustaceans, lepidopteran insects and mytilid molluscs) in species where overlaps did occur there were very high intra-specific distance estimates relative to the other estimates in the familial (or nearest higher taxon) unit (Table 1). Possible reasons for this are discussed briefly below.

In DNA based diet studies that intend to utilize sequence data to assign identity to prey, the size of the amplicon targeted by PCR must necessarily be small to facilitate successful PCR reactions, due to the highly degraded nature of DNA from diet samples (Deagle et al., 2006). Using such a short DNA fragment as we have in this study, it is unsurprising that some species have overlapping divergence within species compared to between species, especially in recently radiated groups or those that are taxonomically difficult and/or unresolved in general. This situation is the same for any DNA-based identification study that intends to use a small DNA fragment, as no small DNA region will be perfect for identifying all species. Situations where there were laboratory errors (either in sample identification or subsequent analysis) would also artificially inflate intra-specific divergence estimates and confound some of these results. Alternatively with no laboratory error, paraphyletic groups that are recognised taxonomically as one group would also inflate intra-specific divergence estimates. Undoubtedly both of these potential errors are likely to be present when obtaining sequence data from GenBank. In general however, this fragment appears to delineate species effectively in the majority of cases and should prove useful for DNA based diet analysis utilizing sequence data for prey identification.

*References:*

Deagle, B. E., J. P. Eveson and S. N. Jarman (2006). Quantification of damage in DNA recovered from highly degraded samples – a case study on DNA in faeces. *Frontiers in Zoology* **3**(11).

Hajibabaei, M., D. H. Janzen, J. M. Burns, W. Hallwachs and P. D. N. Hebert (2006). DNA barcodes distinguish species of tropical Lepidoptera. *Proceedings Of The National Academy Of Sciences Of The United States Of America* **103**(4): 968-971.
